# Psychological Characteristics of Patients with Takotsubo Syndrome and Patients with Acute Coronary Syndrome: An Explorative Study toward a Better Personalized Care

**DOI:** 10.3390/jpm12010038

**Published:** 2022-01-04

**Authors:** Alessandra Gorini, Federica Galli, Mattia Giuliani, Antonia Pierobon, José Pablo Werba, Edvige Palazzo Adriano, Daniela Trabattoni

**Affiliations:** 1 IRCCS Istituti Clinici Scientifici Maugeri di Milano, 20138 Milan, Italy; edvige.palazzoadriano@icsmaugeri.it; 2 Department of Oncology and Hemato-Oncology, University of Milan, 20122 Milan, Italy; 3 Department of Dynamic and Clinical Psychology and Health Studies, Faculty of Medicine and Psychology, Sapienza University of Rome, 00185 Rome, Italy; f.galli@uniroma1.it; 4 Centro Cardiologico Monzino, IRCCS, 20138 Milan, Italy; mattia.giuliani@cardiologicomonzino.it (M.G.); pablo.werba@cardiologicomonzino.it (J.P.W.); daniela.trabattoni@cardiologicomonzino.it (D.T.); 5 Psychology Unit, Istituti Clinici Scientifici Maugeri, IRCCS, Montescano Institute, 27040 Pavia, Italy; antonia.pierobon@icsmaugeri.it

**Keywords:** cardiovascular diseases, Takotsubo Syndrome, Acute Coronary Syndrome, psychological characteristics, personality, health anxiety, emotional triggers

## Abstract

During an acute cardiac event, Takotsubo Syndrome (TTS) and Acute Coronary Syndrome (ACS) apparently share very similar clinical characteristics. Since only a few inconsistent studies have evaluated the psychological features that characterize these different patients, the aim of the present explorative research was to investigate if post-recovery TTS and ACS patients present different psychological profiles. We also investigated whether the occurrence of acute psychological stressful episodes that had occurred prior to the cardiac event could be found in either syndrome. Twenty TTS and twenty ACS female patients were recruited. All patients completed self-report questionnaires about anxiety and depressive symptoms, perceived stress, type-D personality and post-traumatic symptoms. Results showed that only three subscales of health anxiety (i.e., Fear of Death/Diseases, Interference and Reassurance) significantly differed between the two groups, while no differences were found in the other psychological measurements. Moreover, personality traits seem to not be associated with the impact of the cardiac traumatic event. Finally, only TTS patients reported the presence of a significant emotional trigger preceding the acute cardiac event. In conclusion, post-recovery TTS patients differ from ACS patients in their level of concern about their health and in their need of reassurance and information only, probably as a result of the different clinical characteristics of the two illnesses.

## 1. Introduction

In the new era of personalized medicine, the analysis and care of psychological factors acquires a pivotal role in the clinical workflow. On this framework, grounded research has been conducted on the specificity of psychological factors related to heart disease. Of particular interest in this context is the investigation of psychological characteristics of patients affected by different diseases who share very similar clinical symptoms, such as the Takotsubo Syndrome (TTS) and the Acute Coronary Syndrome (ACS). Due to these similarities, TTS has been underdiagnosed for years and only recently researchers have started to pay closer attention to it. Apart from the evident lack of the atherosclerotic component in TTS patients, according to a recent metanalysis, TTS patients have significantly lower left ventricle ejection fraction (LVEF) values on admission compared to ACS patients; however, cardiovascular risks are fewer and the recovery from LVEF is notably higher at both discharge and follow-up in TTS than in ACS. Moreover, there are no significant differences regarding either in-hospital mortality or long-term mortality between the two groups [1]. Furthermore, a peculiar characteristic of TTS is that almost 90% of cases occur in post-menopausal women [2], suggesting that reproductive and hormonal factors play a key role in its development [3]. Moreover, TTS is also observed in men, especially in those on chemotherapy.

Although etiology, epidemiology, and pathophysiology of TTS are still partially unknown, stress and catecholamines seem to be significantly involved in its pathogenesis [4,5,6]. In line with this hypothesis, clinical observation and evidence-based data agree that TTS is often preceded by sudden stressful emotional or physical events occurring a few hours or days before the acute cardiac event [7,8,9]. For this reason, TTS is also known as “broken heart syndrome” or “stress-induced cardiomyopathy” [10]. The role of emotional stressors as frequent triggers for TTS and the sometimes-observed comorbidity with different psychopathologic disorders have been largely discussed in the recent consensus document on the diagnostic workup and management of TTS [8]. However, evidence on the role and the incidence of psychological factors in TTS are sparse, and do not lead to any definitive assumption [11]. Although less frequent, the role of psychological factors as possible contributors to the evolution of cardiac events seems not to be specific to TTS, since it has also been observed in other cardiac diseases, including ACS [12].

Considering the clinical similarities between ACS and TTS, as well as the peculiarities of the latter, we conducted an explorative study to investigate whether: (1) the presence of acute stressful emotional situations preceding the acute cardiac event is more common in TTS than in the ACS patients; (2) TTS differs from ACS in psychological characteristics (e.g., personality, psychopathology) over years after the diagnosis.

## 2. Materials and Methods

### 2.1. Sample

TTS patients previously admitted for their acute episode in a highly specialized cardiac hospital in Northern Italy were called back and asked to participate in the study. Among them, 20 women satisfied the inclusion/exclusion criteria and accepted the invitation to participate. Being rare, the TTS patients were recruited first, then 20 ACS (both STEMI and NSTEMI) matched with them for age, education and time of the diagnosis were enrolled.

The TTS diagnosis was previously performed according to the following Mayo Clinic diagnostic criteria: (1) presence of akinesia or dyskinesia of the apical and/or midventricular segments of the left ventricle with regional wall motion abnormalities that extend beyond the distribution of a single epicardial vessel; (2) presence of sign and symptoms suggesting ACS (i.e., new-onset electrocardiographic abnormalities such as ST-segment elevation and/or T-wave inversion, modest elevation in cardiac troponin levels, and/or typical angina complaints); and (3) absence of obstructive coronary artery disease, pheochromocytoma or myocarditis that could account for the condition.

Exclusion criteria for participating in the study were: (1) age > 85 years; (2) history of more than one acute cardiac episode; (3) current active treatments for cancer or for other life-threating conditions; (4) impaired cognitive abilities. Demographic and clinical characteristics of the samples are reported in Table 1.

All participants were tested during a period of two months from the beginning of recruitment.

The study was approved by the hospital ethical committee and all participants provided informed consent prior to participating in the study.

### 2.2. Patients’ Assessment

Once recruited, all participants underwent a psychological assessment consisting of an open question regarding the occurrence of stressful event(s) at the time of the onset of the disease and of seven self-report psychological validated questionnaires to evaluate depressive symptoms (Beck Depression Inventory—II; BDI-II) [13], state and trait anxiety (State-Trait Anxiety Inventory—Y; STAI-Y) [14], perceived stress (Perceived Stress Scale; PSS) [15], health related anxiety (Health Anxiety Questionnaire; HAQ) [16], the impact of the traumatic event (if any) (Impact of Event Scale; IES) [17], and Type-D personality traits (Type-D Scale; DS-14) [18]. All the questionnaires were aimed at assessing the psychological status of the patients after a medium–long time from the acute event, to catch stable psychological characteristics that may have been related to the acute phase. The description of the questionnaires is presented below:

#### 2.2.1. Beck Depression Inventory—II (BDI-II)

The BDI-II is widely used to evaluate the severity of depressive symptoms in adult and adolescent patients. This consists of 21 items with four response options, ranging from 0 (i.e., “Not Present”) to 3 (i.e., “Severe”). It provides four symptoms’ categories based on the obtained score: 0–13 (minimal depression); 14–19 (mild depression); 20–28 (moderate depression); and 29–63 (severe depression). The internal consistency was described as around 0.92 and the test–retest reliability is 0.93 [19].

#### 2.2.2. State-Trait Anxiety Inventory (STAI-Y)

The STAI-Y is widely used to measure trait and state anxiety. Y form is the most widespread version and comprises of 20 items for assessing trait anxiety (e.g., “I worry too much over something that really doesn’t matter”) and 20 for state anxiety (e.g., “I am tense”, “I am worried” or “I feel calm”). All items are rated on a 4-point scale, from “Almost Never” to “Almost Always” and no cut-off points are used: the higher the total score, the more severe the anxiety symptoms. Internal consistency coefficients for the scale have ranged from 86 to 95; test–retest reliability coefficients have ranged from 65 to 75 over a 2-month interval [14]. Test–retest coefficients for this measure in the present study ranged from 69 to 89.

#### 2.2.3. Perceived Stress Scale (PSS)

The PSS is the most widely used self-report questionnaire for measuring distress perception. The items evaluate the frequency of feelings and thoughts related to distress perception with a score ranging from 0 to 40. The score interpretation is based upon three values categories: 0–13 (i.e., low stress); 14–26 (i.e., moderate stress); and 27–40 (i.e., high stress). Reliability values for Cronbach’s alphas in the Italian version [20] are all from acceptable to good and range from 0.74 for the aggregate score, 0.72 for the “positive stress” subscale and 0.84 for the “negative stress subscale.

#### 2.2.4. Health Anxiety Questionnaire (HAQ)

The HAQ is a self-report questionnaire, which consists of 21 items describing health anxiety related symptoms. This consists of four subscales, which measure health worry and preoccupation, fear of illness and death, reassurance-seeking behavior and interference with life. The original version of the HAQ has shown good psychometric properties, and its Italian version [21] has shown adequate internal consistency (α N 77 for all subscales), temporal stability (r = 89) and construct validity.

#### 2.2.5. Impact of Events Scale (IES)

The IES is a 15-item self-report questionnaire, which is widely used to evaluate event-specific distress. This comprises of two subscales: intrusiveness (i.e., frequency of intrusive cognition associated with a specific stressor); and avoidance (i.e., frequency of avoidant behaviors associated with a specific stressor). Each item is scored through a 4-point scale, ranging from 0 (i.e., not at all) to 3 (i.e., often). In the present study, participants were asked to consider an acute cardiac event as the reference event. The questionnaire has an adequate internal consistency (alpha = 0.80–0.93 for the intrusion; alpha = 0.73–0.84 for avoidance) and high test–retest reliability (r = 0.93) [22].

#### 2.2.6. Type-D Scale (DS-14)

The DS-14 is a brief self-report questionnaire, which is used worldwide to evaluate type D personality traits: negative affectivity (NA) and social inhibition (SI). The DS-14 comprises of 14 items, each evaluated on a scale between 0 (i.e., false) and 4 (i.e., true). This provides two separate scores for NA and SI, each ranging from 0 to 28. A cut-off score of ≥10 means the presence of a maladaptive personality trait. Gremigni and Sommaruga [23] highlighted the good psychometric properties of the Italian version of DS-14, and they recommend its use in psychological screening for clinical research.

Finally, the data about the presence of a stressful life-event temporally linked to the acute cardiac event and potentially related to the occurrence of both TTS and ACS were collected from the clinical charts written at the time of hospitalization.

### 2.3. Statistical Analyses

All statistical analyses were performed using the IBM SPSS 26.0 software. Normality was checked through the Shapiro–Wilk Test. Since no variables followed a normal distribution, non-parametric tests were used. The Mann–Whitney U Test was run to evaluate the differences between groups in demographic (i.e., Age and Education), clinical (i.e., Menopausal Age and Years from the Event) and personality variables (i.e., DS-14 and EPQR-S). Spearman’s Rho coefficient was used to perform the correlations analyses between personality traits (i.e., DS-14) and the perceived impact of the event (i.e., IES).

## 3. Results

### 3.1. Demographic and Clinical Characteristics

TTS and ACS groups did not differ in age, years of education, menopausal age and the period of time elapsed between the acute cardiac event and the time of assessment (see Table 1).

### 3.2. Occurrence of Stressful Events Related to the Acute Clinical Event

Only TTS patients reported significant stressful events in the week before the acute cardiac illness that caused hospitalization. In particular:

Six patients reported the death of a relative, a friend or a significant one;
Three patients reported a serious quarrel with a relative or a friend;
Six patients reported an assault suffered or witnessed (e.g., snatch or domestic violence);
Three patients reported an acute stress related to specific work or family issues.

The two remaining TTS patients were involved in specific events: a building collapse and a car accident.

### 3.3. Psychological Assessment: Questionnaires

The TTS patients showed lower levels of concern about their own death or about the possibility of contracting diseases (fear of death/diseases subscale) (*p* < 0.05) and less interfering thoughts regarding the acute cardiac episode compared to the ACS patients (interference subscale) (*p* < 0.001). Moreover, compared to the ACS sample, the TTS patients showed a greater need to seek reassurances from friends and family, as well as a greater need of receiving information about their health when they feel alarming body signals (reassurance subscale) (*p* < 0.05). None of the other examined psychological variables differed between the two groups. These results are reported in Table 2, Table 3 and Table 4.

### 3.4. Psychological Assessment: Personality Traits

Forty percent (i.e., 12) and 25% (i.e., 5) of patients with TTS showed, respectively, NA and SI scores over the cutoff point. Fifty-five percent (i.e., 11) and 35% (i.e., 7) of patients with ACS showed, respectively, NA and SI scores over the cutoff point. No significant correlations between Type-D personality traits (i.e., NA and SI) and the items of the subscale of the IES (i.e., avoidance, intrusiveness and the total score) were found in the TTS or in the ACS groups.

## 4. Discussion

The present explorative study had two main aims: (1) to investigate if the presence of acute stressful emotional situations preceding the acute cardiac event is more common in TTS than in the ACS ones; (2) to investigate if TTS patients have specific psychological traits compared to ACS patients. Analyzing the correlation between personality traits and the perceived impact of the cardiac event, we were also interested in verifying if there was an association between the two, since personality is known to influence the way in which individuals cope with stressful situations. Such outcomes are supposed to vary across sociodemographic groups and characteristics. However, given that sociodemographic characteristics are not statistically different across the two patient groups, despite the low number of participants, the two groups can be feasibly compared without controlling for sociodemographic variation.

Regarding the triggering effect of emotional stimuli, our data show that all the TTS patients, although not the ACS ones, reported the occurrence of a significant stressful event during the week before the hospitalization. According to a recent TTS consensus paper [8], all the events reported by our sample may be classified as emotional triggers, confirming previous findings showing that TTS in women is often related to emotional stressful events and differs in men, for whom physical triggers are more frequent [9], and in ACS patients, where the occurrence of a stressful triggering experience is low [24]. The occurrence of an additional acute stressful life-event occurring in individuals with a physical predisposition to TTS who have exceeded their ability to cope due to the cumulative burden of chronic stress and life events (allostatic overload) has been recently outlined as a possible mechanism related to TTS [25]. Deepening these aspects by a psychological assessment is crucial for personalizing care in clinical settings. Also differentiating between emotional and physical triggers of TTS would require a psychological evaluation to accurately recognize if the physical trigger did not burden the emotional side of the patient.

Several studies have investigated how stress can favor the onset of TTS from a biological point of view. According to Delmas and colleagues, the pathophysiology of TTS may include an autonomic nervous system dysfunction, that consists in a “downregulation of autonomic modulation by the parasympathetic tone leading to an excessive response to acute sympathetic nervous system stimulation, with fast and complete recovery after the stimulation itself” [26]. A recent neuroimaging study also suggests an impairment in emotion regulation patterns for TTS patients [27]. From the behavioral side, an excessive response to a stressful situation may be due to a scarce ability to cope with stress. Similarly, poor coping abilities may be responsible for a persistent state of stress. According to these considerations, we could expect to find a higher level of perceived stress in TTS compared to ACS, even some time after the acute event, although this was not the case in the present study since we did not find any significant difference between the two groups.

In the current research, TTS and ACS patients also obtained similar scores to the BDI-II and the STAI-Y questionnaires (both on trait and state) showing the absence of actual clinically relevant depressive and anxious symptoms in both groups. Nevertheless, our findings are different from those obtained in other studies showing a higher prevalence of anxiety, or of both anxiety and depression, in TTS patients compared to the ACS ones [26,28,29], or an increased presence of depression in the years following the ACS event [30]. These differences may be explained with the methodological differences that differentiate our study from the others, including the numerosity of the samples (the previous ones were obtained from very large databases and samples), the presence of men, and the questionnaires used for the assessment. Moreover, we assessed the psychological status of the patients after months or years after the acute cardiac event with the purpose of verifying whether these cardiac illnesses may be related to different psychological/psychopathological traits in the long-term, finding negative results. These heterogeneous observations and the poor amount of available literature investigating the presence of anxiety and/or depression in TTS and ACS patients suggest the need of a deeper investigation of this issue in future studies. Moreover, further research is needed to assess the likely interplay of emotional regulation patterns, psychopathology and the way of coping with perceived stress in TTS patients compared to ASC patients.

The only significant differences between the two groups of patients that emerged from this explorative study regard the three subscales of the HAQ. TTS patients show less concern about dying or contracting serious illnesses than ACS patients. Moreover, TTS patients have a greater need to seek reassurances from friends and family, as well as a greater need to receive information about their health when they feel alarming body signals, compared to ACS patients. Conversely, the ACS patients manifest a higher fear of death or the development of other diseases, as well as higher intrusive thoughts about the cardiac event compared to TTS patients. Far from finding exhaustive explanations for these results, we argue that the ACS patients’ fears and worries are related to the fact that they usually receive a worst prognosis, require coronary revascularization procedures, longer hospitalization time and more intensive care, and are often forced to significantly change their lifestyle compared to TTS patients, whose acute cardiac episode has a complete resolution within a few weeks without leaving any specific or severe consequence. In fact, even though the exact cause of health anxiety is unknown, past experiences with serious illness may be a possible risk factor and a reasonable explanation for it [31].

Nevertheless, compared to ACS, TTS is rare and still poorly understood, and that should be the reason why TTS patients require more reassurances and information. These different experiences can be so significant they have a lasting impact, even in the long-term. However, these are only partial explanations and further studies are necessary to better understand the obtained results.

Finally, personality may have a role in influencing cardiac activity, as it is related to the way people usually cope with and respond to daily stressful situations. In particular, Type-D personality (i.e., “Distressed” personality, featured by NA and SI) has been historically associated with cardiovascular disorders [32]. However, neither NA nor SI subscales significantly differed between TTS and ACS patients, and no significant correlations were found with any of the IES scale, suggesting that these personality traits are not related to the avoidance behavior, or the intrusive thoughts related to the acute cardiovascular event. Our results regarding personality traits in TTS patients are part of an open debate, since there are only a few conflicting studies of TTS, as opposed to other cardiac diagnoses, such as ACS [11]. Thus, further studies are needed in order to understand if and how personality traits are related to TTS.

Although this study has the advantage of having addressed the potential differences in psychological characteristics of TTS compared to ACS patients, which is an often neglected issue in the TTS literature, it has also some important limitations. First of all, the observed samples are small. Nevertheless, numerosity is not so different from that considered in the majority of previous studies, mainly due to fact that TTS is still a rare and/or underdiagnosed disease [33,34,35,36]. The second limitation, linked to the difficulties in finding TTS patients, is related to the time of assessment that varied a lot among subjects and between the two selected groups. Nevertheless, since we were interested in observing the long-term psychological characteristics of patients, and the difference among the TTS and ACS groups was not statistically significant, we considered the two groups comparable. As a third limitation, at the time of the study, we did not have access to some relevant patients’ data related to the acute event, including, but not limited to, the severity of clinical presentation, the length of hospital stay, and the occurrence of adverse events during short-term follow up. Such data could have had an impact on the post-event psychological status and need to be considered in future studies.

Nevertheless, inasmuch as we are aware of the described shortcoming, this explanatory study proposes interesting preliminary findings.

## 5. Conclusions

In conclusion, we can affirm that post-recovery TTS patients do not appear to have different psychological profiles as compared to ACS patients, except for the fact that they are less worried about their health, probably due to the clinical characteristics of their illness (i.e., a better prognosis and a lower impact on their life compared to ACS). Anyway, the limited number of our samples, mainly due to the fact that TTS is an under-diagnosed and quite rare disease, suggests the need of further psychological evaluations, both in the acute phase of the illness and in the long-term, to provide better patient care focused not only on the clinical aspects of the disease, but also on its psychological implications.

## Figures and Tables

**Table 1:** Demographic and clinical characteristics of TTS and ACS patients.

		TTS (*N* = 20)		ACS (*N* = 20)		Z	r	*p*-Value
		Mean	SD	Mean	SD			
Age		68.00	8.67	69.25	9.19	−0.637	−0.142	0.524
Education (years)		12.50	4.11	10.40	3.97	−1.821	−0.407	0.069
Menopause (age)		49.95	4.06	48.13	4.92	−0.999	−0.223	0.318
Years from the Event		5.05	3.54	9.38	9.10	−1.181	−0.264	0.237
		N	%	N	%	Chi-square	*p*-value	
Marital Status	Single	0	0.0	3	15.0	5.926	0.205	
	Unmarried Couple	1	5.0	0	0.0			
	Married Couple	16	80.0	11	55.0			
	Divorced	1	5.0	2	10.0			
	Widowed	2	10.0	4	20.0			
Induced Menopause	Yes	3	15.8	5	25.0	0.781	0.377	
	No	17	84.2	15	75.0			

**Table 2:** Psychological characteristics of TTS and ACS patients.

		TTS (*N* = 20)		ACS (*N* = 20)		Z	r	*p*-Value
		Mean	SD	Mean	SD			
BDI-II		9.53	8.57	9.75	6.30	−0.338	−0.076	0.753
STAI-Y	State Anxiety	37.90	10.12	38.50	9.55	−0.271	−0.061	0.786
	Trait Anxiety	38.30	11.00	40.30	7.45	−0.433	−0.097	0.665
PSS		16.60	8.39	17.85	6.09	−0.122	−0.027	0.903
HAQ	Fear of Death/Diseases	4.30	3.20	6.10	3.63	−2.167	−0.485	0.030
	Worries about Health	5.40	3.02	5.30	3.47	−0.082	−0.018	0.935
	Interference	0.65	1.39	2.15	1.50	−3.703	−0.828	<0.001
	Reassurance	2.60	1.47	1.60	2.39	−2.504	−0.560	0.012
	Total	12.95	7.23	15.15	8.43	−1.238	−0.277	0.216
IES	Intrusivity	9.65	9.24	12.28	7.95	−1.244	−0.278	0.213
	Avoidance	10.55	11.07	14.28	8.71	−1.305	−0.292	0.192
	Total	20.20	17.72	25.16	15.63	−1.069	−0.239	0.285
DS-14	Negative Affectivity	9.65	7.36	10.15	5.842	−0.488	−0.109	0.626
	Social Inhibition	5.40	4.75	6.95	6.030	−0.750	−0.168	0.454

BDI-II, Beck Depression Inventory-II; STAI-Y, State-Trait Anxiety Inventory Y-form; PSS, Perceived Stress Scale; HAQ, Health Anxiety Questionnaire; IES, Impact of the Event Scale; DS-14, Type-D Scale.

**Table 3:** Correlations between the Impact of Event Scale (IES) and the Type-D Scale (DS-14) in patients with TTS.

	IES Intrusivity	IES Avoidance	IES Total Score
DS-14 Negative Affectivity	r = 0.250 *p* = 0.229	r = 0.312 *p* = 0.129	r = 0.366 *p* = 0.072
DS-14 Social Inhibition	r = 0.371 *p* = 0.068	r = 0.157 *p* = 0.454	r = 0.261 *p* = 0.208

**Table 4:** Correlations between the Impact of Event Scale (IES) and the Type-D Scale (DS-14) in patients with ACS.

	IES Intrusivity	IES Avoidance	IES Total Score
DS-14 Negative Affectivity	r = −0.096 *p* = 0.705	r = 0.200 *p* = 0.425	r = 0.068 *p* = 0.781
DS-14 Social Inhibition	r = 0.067 *p* = 0.790	r = 0.231 *p* = 0.357	r = 0.004 *p* = 0.987

## Data Availability

The database is available at: https://doi.org/10.5281/zenodo.5723798 (accessed on 25 November 2021).
